# Quinone Based Materials as Renewable High Energy Density Cathode Materials for Rechargeable Magnesium Batteries

**DOI:** 10.3390/ma13030506

**Published:** 2020-01-21

**Authors:** Jan Bitenc, Tjaša Pavčnik, Urban Košir, Klemen Pirnat

**Affiliations:** 1 Department of Materials Chemistry, National Institute of Chemistry, Hajdrihova 19, 1000 Ljubljana, Slovenia; tjasa.pavcnik@ki.si (T.P.); klemen.pirnat@ki.si (K.P.); 2 Faculty of Chemistry and Chemical Technology, University of Ljubljana, Večna pot 113, 1000 Ljubljana, Slovenia; urban.kosir96@gmail.com

**Keywords:** magnesium rechargeable batteries, organic materials, quinones, electrolyte effect, ex-situ IR spectroscopy

## Abstract

Organic cathode materials are promising cathode materials for multivalent batteries. Among organic cathodes, anthraquinone (AQ) has already been applied to various metal‒organic systems. In this work, we compare electrochemical performance and redox potential of AQ with 1,4-naphthoquinone (NQ) and 1,4-benzoquinone (BQ), both of which offer significantly higher theoretical energy density than AQ and are tested in two different Mg electrolytes. In Mg(TFSI)_2_-2MgCl_2_ electrolyte, NQ and BQ exhibit 0.2 and 0.5 V higher potential than AQ, respectively. Furthermore, an upshift of potential for 200 mV in MgCl_2_-AlCl_3_ electrolyte versus Mg(TFSI)_2_-2MgCl_2_ was confirmed for all used organic compounds. While lower molecular weights of NQ and BQ increase their specific capacity, they also affect the solubility in used electrolytes. Increased solubility lowers long-term capacity retention, confirming the need for the synthesis of NQ and BQ based polymers. Finally, we examine the electrochemical mechanism through ex situ attenuated total reflectance infrared spectroscopy (ATR-IR) and comparison of ex situ cathode spectra with spectra of individual electrode components. For the first time, magnesium anthracene-9,10-bis(olate), a discharged form of AQ moiety, is synthesized, which allows us to confirm the electrochemical mechanism of AQ cathode in Mg battery system.

## 1. Introduction

Push towards electromobility and ongoing transfer from fossil fuel-based energy systems towards renewable energy is steadily increasing the demand for ever more and better energy storage technologies. Batteries, as one of the most prominent energy storage devices, should provide a solution to these arising needs. While Li-ion batteries—currently the most mature battery technology—are dominating the market, they are slowly reaching their thermodynamic limitations. In addition, concerns are being raised about the future supply and environmental sustainability of certain elements (Co, Li) used in contemporary Li-ion batteries, especially for certain resource-poor countries, such as the EU. This is motivating researchers to search for alternative battery technologies that would, at the same time, offer comparable or higher energy densities and would also be based exclusively on sustainable materials. There are many commercially available alternative battery technologies. However, the established systems, such as Pb-acid, Ni-MH, and Na/NiCl_2,_ cannot compete in terms of the energy density with Li-ion. Thus, research focus has been moved to the development of novel high-energy battery systems. For anode materials, there are several options among abundant metals such as Na, K, Mg, Ca, and Al, which are all among the ten most abundant elements in the Earth’s crust [1]. However, both Na and K metals are prone to dendrite formation [2]. On the other hand, Mg has already exhibited non-dendritic metal deposition morphology [3] and exemplifies lower surface diffusion barriers as alkali metals [4]. However, some recent reports have shown the formation of Mg dendrites under certain conditions [5,6]. Relatively high density of Mg metal compared to alkali metals means that the volumetric energy is almost twice as high as in case of Li metal. Standard redox potential of Mg metal is not as low as that of Li metal, which results in the overall lower voltage of the Mg battery. One of the major disadvantages of multivalent battery systems is a relatively small number of available electrolytes, which are additionally limited in certain properties, such as operating voltage window, conductivity, thermal stability [7].

Finding a suitable host material for the cathode side is much more challenging. While there is a wide choice of materials capable of intercalating Li^+^ ions and even Na^+^ ions, the intercalation of K^+^ ions is much more challenging. There are only a handful of inorganic materials that enable intercalation of Mg^2+^ ions, mainly chalcogenide-based compounds and Prussian blue analogs. Main intercalation limitations are connected with the slow solid-state diffusion and strong interactions between bivalent Mg^2+^ ion and host material, which often lead to irreversible side reactions [8]. Hence, a conceptual shift of cathode development is required to speed up the development of post-Li-battery technologies. Organic cathode materials can accommodate different cations and their performance has been already demonstrated with various metal counterions (Na, K, Mg, Al, Zn, Pb, Ni) [9,10,11,12,13,14]. Their synthesis is typically performed at lower temperatures and from abundant materials, which could significantly lower the environmental footprint of future battery systems. The redox potential of organic compounds can also be fine-tuned to a certain degree through the introduction of various functional groups. Although care should be taken that the introduced functional groups do not increase the molar mass too much since an increase in molecular weight lowers the specific capacity of active material. The biggest downside of organic materials is the solubility of simple organic molecules as it may lead to rapid capacity decay during cycling. Normally, this can be avoided by the preparation of insoluble polymers, grafting onto solid particles, or using ion-selective separators. However, the last approach is mostly limited only to Li systems due to the lack of selective separators in other battery systems. Grafting onto solid particles and preparation of polymers both come at a cost of decreased capacity. Grafting introduces electrochemically inactive solid particles and polymerization increases the molecular weight of the electroactive group due to the introduction of a linker. However, the use of relatively small linker groups or even direct cross-coupling without any linker makes polymerization the most effective for practical applications.

Anthraquinone (AQ), its derivatives, and AQ based polymers have been extensively studied in Li [15,16,17,18], but also in other mono- and multivalent systems, such as Na [9], K [10], Mg [11,19,20], and Al [12]. Its electrochemical redox potential in Li electrolyte is around 2.2 V, which fits well within the potential window of state-of-the-art multivalent electrolytes, where the relatively low oxidative limit is a common limitation. Although cell voltage of Li‒AQ is around 2.2 V, it is significantly lower for Mg‒AQ (1.5 V) and even much lower for Al‒AQ (0.8 V), presuming that cathode potential does not change in multivalent electrolytes. Relatively low voltage can be compensated by the high capacity of the metal anode. However, it still presents a challenge for practical applications, because energy loss due to voltage hysteresis is much larger as in comparable high-voltage cells. This makes a push for high voltage organic cathodes necessary. Fortunately, the diversity of organic compounds offers several interesting groups with higher voltage and capacity as AQ. Most obvious next-generation materials are those based on naphthoquinone and benzoquinone groups, which belong to the same conjugated carbonyl group of materials. Although nitroxyl radicals typically exhibit even higher potential as conjugated carbonyls, we do not consider them as practical alternatives. They are p-type materials and require a counter anion for a reversible reaction. Anions typically come from electrolyte, which significantly increases the amount of electrolyte needed, greatly decreasing the cell energy density.

Herein, in this work, we focus on the performance of three quinone-based organic compounds: Anthraquinone (AQ), 1,4-naphthoquinone (NQ), and 1,4-benzoquinone (BQ). We study their performance in two common Mg electrolytes and investigate electrochemical mechanism through ex situ IR spectroscopy. Both NQ and BQ have already been, to a certain extent, studied in the Li system [21]. However, their application is much more limited due to high solubility in the battery electrolytes. Synthesis of polymers from benzoquinone and naphthoquinone is more complicated due to their higher redox-potential, which complicates polymerization procedures typically used for the preparation of AQ based polymers [22,23]. Nevertheless, certain synthesis procedures have given polymers with relatively good electrochemical performance for sulfur-based BQ polymers [22,24], but capacities close to theoretical ones are still to be achieved. NQ molecule has a theoretical capacity of 339 mAh/g and redox potential of 2.4 V vs. Li, while capacity and redox potential of benzoquinone are even higher, 496 mAh/g and 2.7 V vs. Li.

Higher redox potentials make NQ and BQ materials especially interesting for application in multivalent batteries (Table 1). While the theoretical energy density of 345 Wh/kg for Mg–AQ cell is relatively low, an Mg–BQ cell can offer more than two times higher energy density of 810 Wh/kg and Mg–NQ cell reaches somewhere in between with 500 Wh/kg (Table 1). The difference is even larger for Al cells, where the energy density of Al‒BQ is almost three times higher than the energy of Al‒AQ. Such an increase in energy density is well-worth the investigation, especially given the fact that Mg metal anode‒organic batteries are a more realistic possibility than Li metal anode‒organic batteries due to the nature of Mg metal deposition.

## 2. Materials and Methods

### 2.1. Material Preparation

Anthraquinone (99% Fluka), 1,4-naphthoquinone (96.5% Fluka), 1,4-benzoquinone (99+%, Sigma), anhydrous MgCl_2_ (ultra-dry 99.99% Alfa Aeasar), anhydrous AlCl_3_ (99.985% Alfa Aeasar), carbon black Printex XE2 and polytetrafluoroethylene (PTFE) water dispersion (60% in water, Sigma Aldrich) were all used as received. Mg(TFSI)_2_ (Solvionic 99.5%) was dried for at least 24 h at 250 °C in vacuum before use. Dimethoxyethane (DME) (99% Honeywell) was dried with molecular sieves for several days, mixed with Na/K alloy (approx. 1 mL/L) overnight and afterwards, fractionally distilled. Anthracene-9,10-diol was prepared by the reduction of anthraquinone in dimethylsulfoxide with NaBH_4_. Afterwards, anthracene-9,10-diol was dissolved in tetrahydrofuran and reacted with Bu_2_Mg in heptane. The reaction mixture was dried to obtain brick red product magnesium anthracene-9,10-bis(olate) coordinated with tetrahydrofuran solvent. Electrolytes were prepared by adding the appropriate amount of salts into measuring flasks, mixing overnight, and finally, diluting them up to the mark to obtain 0.6 M Mg(TFSI)_2_-2MgCl_2_ and 0.6 M MgCl_2_‒AlCl_3_ in DME.

### 2.2. Electrochemical Characterization

Active materials were mixed with Printex XE2 carbon black and PTFE binder in 60:30:10 weight ratio. All the components and isopropanol were added into ball mill jar and homogenized for 30 min on Retsch PM100 at 300 rpm. Afterwards, composite was transferred to an agate mortar where it was slowly mixed until chewing gum like texture was obtained. Composite was then rolled in between a glass plate and a sheet of baking paper. Afterwards 12 mm self-standing electrodes were cut, dried, and transferred into the glovebox. The loading of active material was around 2 mg/cm^2^. To prevent sublimation 1,4-benzoquinone composite was dried at room temperature and not exposed to vacuum during glovebox transfer. Cells were assembled with one layer of glassy fiber separator and approximately six drops of electrolyte. Mg foil (Changsha Rich Nonferrous metals, 99.95%, 0.1 mm) was polished with P1200 sandpaper inside the glovebox before assembly. On the cathode side graphite disc current collectors were used to prevent corrosion of stainless-steel plunger. Electrochemical testing of the cathodes was performed under galvanostatic mode with VMP3 potentiostat from Bio Logic S. A. in Swagelok type cells.

### 2.3. IR Spectroscopy

IR characterization was performed inside the glovebox using ATR-IR Alpha II (Bruker) with Ge crystal. Measurements were performed in the range from 4000 to 600 cm^−1^ with a resolution of 4 cm^−1^. Cathodes for ex-situ measurements were charged or discharged with 20 mA/g to achieve higher capacity utilization. Afterwards, cathodes were removed from the graphite discs and their IR spectra were measured. In the second step, cathodes were briefly washed with a few ml of DME to remove electrolyte, no extensive washing was performed to limit the dissolution of active material.

## 3. Results and Discussion

**Figure 1:**
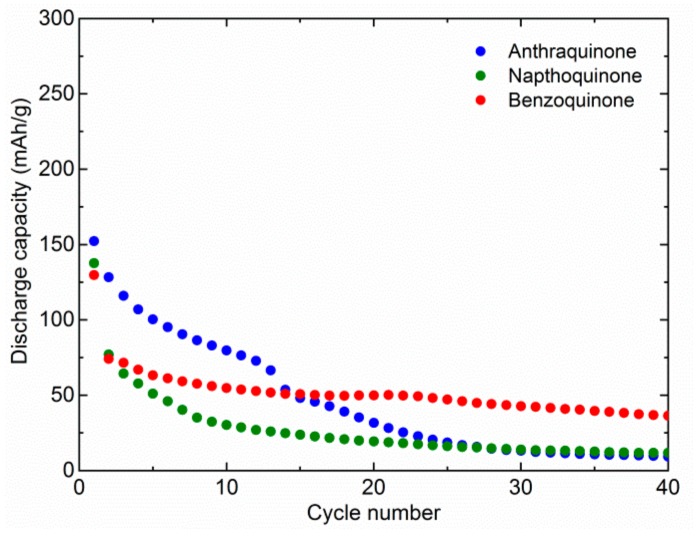
Discharge capacity of different organic cathode materials in MTC electrolytes. All materials were cycled at 100 mA/g. Anthraquinone, 1,4-naphthoquinone, and 1,4-benzoquinone were cycled in different voltage windows 0.5–2.5, 0.75–2.75, and 1.0–3.0 V, respectively.

The electrochemical activity of AQ, NQ, and BQ was compared in two common Mg electrolytes, 0.6 M Mg(TFSI)_2_-2MgCl_2_ (MTC) and 0.6 M MgCl_2_-AlCl_3_ (MAC) in DME, with the focus on MTC electrolyte, which, according to our experience, offers optimum electrochemical cycling for quinone-based materials [11]. Both electrolytes are based on commercially available salts and have been extensively tested in the literature [25,26]. First, all the materials were tested at a relatively high specific current of 100 mA/g (Figure 1). In the first cycle, we observed relatively similar capacities. AQ delivers 152 mAh/g, NQ 138 mAh/g, and BQ 130 mAh/g. This means that although the theoretical capacity is much higher for NQ and BQ, it is difficult to utilize their full capacity. Therefore, theoretical capacity utilization is 59%, 41%, and 26% for AQ, NQ, and BQ, respectively. Rapid dissolution means that part of the material is dissolved almost instantly and cannot be utilized during the first discharge. In the following cycles, the capacity of the AQ starts to drop quite gradually and reaches 80 mAh/g in the 10th cycle. Afterwards, it fades more rapidly and stabilizes around 10 mAh/g, around 30th cycle. The starting capacity of NQ faded more rapidly and retained only 30 mAh/g after 10 cycles. The following capacity fade of NQ was more gradual, and after 40 cycles, a capacity of only 12 mAh/g was retained. The initial capacity fade of BQ was similar to NQ, but it stabilized faster at around 50 mAh/g. Although initial capacity utilization of AQ is the highest, its long-term retention is similar to NQ and BQ. However, it takes much longer for the majority of dissolution to occur.

**Figure 2:**
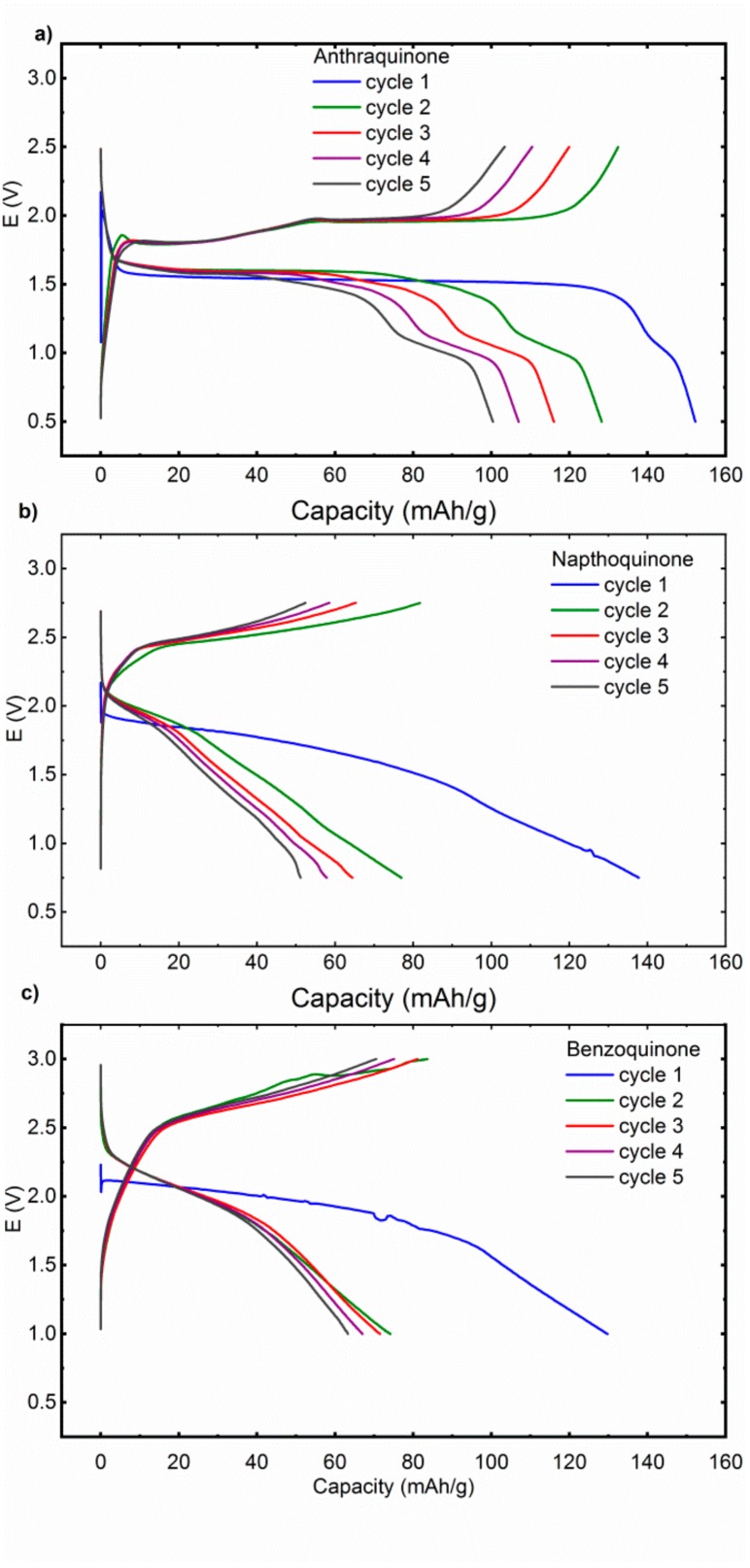
First five discharge cycles of (**a**) AQ, (**b**) NQ, and (**c**) BQ at a specific current of 100 mA/g in MTC electrolyte. AQ, NQ, and BQ were cycled in different voltage windows 0.5–2.5, 0.75–2.75, and 1.0–3.0 V, respectively.

Cell voltage of Mg-organic batteries can be assessed from the difference between Li metal and Mg metal redox potentials under the presumption that the potential of the organic redox group does not change. However, there is a possibility that electrolytes influence the redox potential of organic groups. Such behavior was already observed in beaker cell studies in dimethylformamide perchlorate based electrolytes [27] and also in certain cases with Mg-organic battery cells [11]. Mg‒AQ cell exhibits in the first cycle a discharge potential of 1.55 V, which is in later cycles slightly upshifted for approximately 50 mV (Figure 2). There is also a short second plateau at 1.0 V, which becomes more pronounced in the later cycles. In the charge, there are two well-formed plateaus at 1.8 and 1.95 V. NQ exhibits less defined plateaus, in the first discharge there is a long sloping plateau centered around 1.75 V with a long almost capacitance like tail till the 0.75 V cut-off. In following cycles, the redox plateau becomes even shorter and less defined with an increasing contribution of the capacitance like tail, consistent with the dissolution of active material. Charge displays similar behavior with a long sloppy charge plateau at approximately 2.6 V. Discharge curves of Mg‒BQ cell are similar to NQ with a sloped discharge plateau which gradually ends up in the capacitance like tail. In the first discharge the plateau is centered around 1.9 V and later moves up to 2.0 V. Overall it means that some deviations from the theoretically predicted discharge voltages are observed, but no significant upshift of the redox potential can be observed when moving from Li electrolyte to MTC electrolyte.

**Figure 3:**
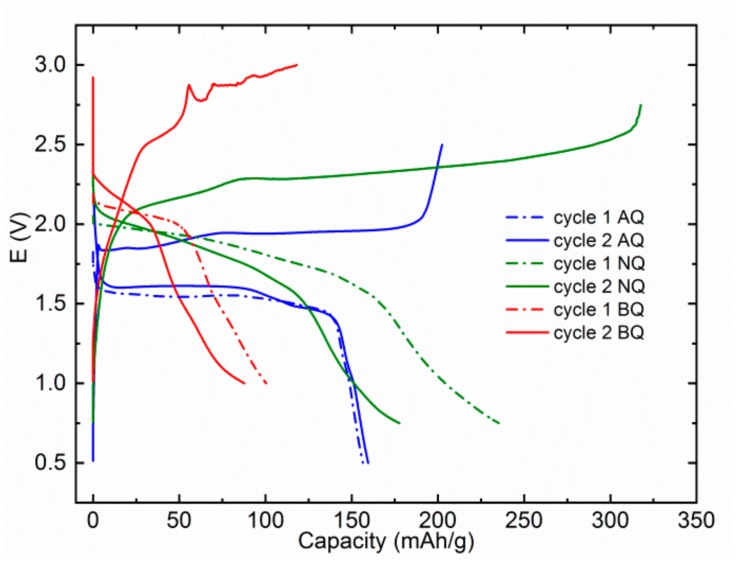
First two discharge cycles for AQ (blue), NQ (green), and BQ (red) at a specific current of 20 mA/g in MTC electrolyte. AQ, NQ, and BQ were cycled in different voltage windows 0.5–2.5, 0.75–2.75, and 1.0–3.0 V, respectively.

Further investigation of MTC electrolytes was performed at lower current rate of 20 mA/g. The idea was to see if relatively low capacities observed in previous tests were connected mainly with dissolution or also poor electrochemical accessibility (inability to transport ions or electrons to electroactive groups) of the active material. All three materials exhibit decreased efficiency of cycling with significantly higher overcharge, which can be attributed to side reactions of electrolytes and shuttling of redox-active compounds. AQ exhibits slightly higher capacity of first discharge at 156 mAh/g and even a slight increase in the second cycle to 160 mAh/g (Figure 3). The biggest difference is observed with NQ, which has a discharge capacity of 235 mAh/g in the first discharge and 178 mAh/g in the second discharge, which is a 70% increase over capacity observed in the first discharge at 100 mA/g. This observation points to the fact that poor electrochemical accessibility was a limiting factor in the previous test. There is no capacity increase in the case of BQ in the first cycle, on contrary, a small capacity decrease is observed pointing to the fact that the dissolution of active material is the main cause for the low capacity of BQ. An additional feature that we observed is the decomposition of the electrolyte, which starts at approximately 2.8 V. This underlines an important observation that although electrolytes exhibit high oxidative stability above 3 V in the cyclic voltammetry or linear sweep voltammetry tests on metal working electrodes, stability might be significantly lower in the presence of practical cathode materials. This instability might be less visible at relatively high specific currents but becomes much more apparent at lower specific currents/C-rates. This is an important limitation, especially for Mg electrolytes, which are mostly based on ether solvents that have significantly lower oxidative limits than carbonate solvents, typically used in Li-ion batteries.

**Figure 4:**
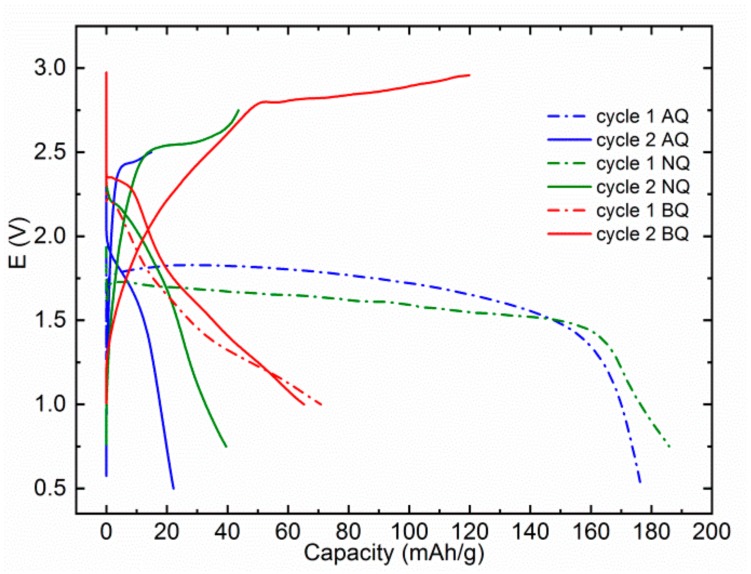
First two discharge cycles for AQ (blue), NQ (green), and BQ (red) at a specific current of 100 mA/g in MAC electrolytes. AQ, NQ, and BQ were cycled in different voltage windows 0.5–2.5, 0.75–2.75, and 1.0–3.0 V, respectively.

Although no voltage upshift was reported in the MTC electrolyte, some older literature reports and recently published battery studies show a considerable upshift of AQ redox potential in AlCl_3_ containing Al and Mg electrolytes [11,12,28,29]. Thus, we wanted to check if the effect could be generalized also on other electroactive groups in MAC electrolyte, which contains AlCl_3_. The effect could be of significant importance since an upshift of voltage for only 0.2 V would mean an increase of energy density for 46 Wh/kg in the case of Mg‒AQ and 81 Wh/kg in the case of Mg‒BQ. AQ moiety in MAC electrolyte displays a discharge potential of 1.75 V in the first discharge (Figure 4). Its plateau is even higher than the first discharge plateau of NQ in MAC at 1.6 V. Interestingly NQ displays a better-defined plateau in the MAC electrolyte than in MTC. In the second cycle, the discharge plateau of NQ moves up to 2.0 V, but the shape of the curve is changed from a well-defined flat plateau to the sloped curve with a long capacitance tail. BQ exhibits a sloped discharge plateau in both first and second discharge cycles at 2.2 and 2.3 V, respectively. However, the discharge capacity fade from the first to second cycle in MAC electrolyte is even bigger and we obtained less than 70 mAh/g in the second discharge. This could be caused by a lower concentration of salts in MAC electrolytes, which might increase the solubility of organic compounds in this electrolyte.

**Figure 5:**
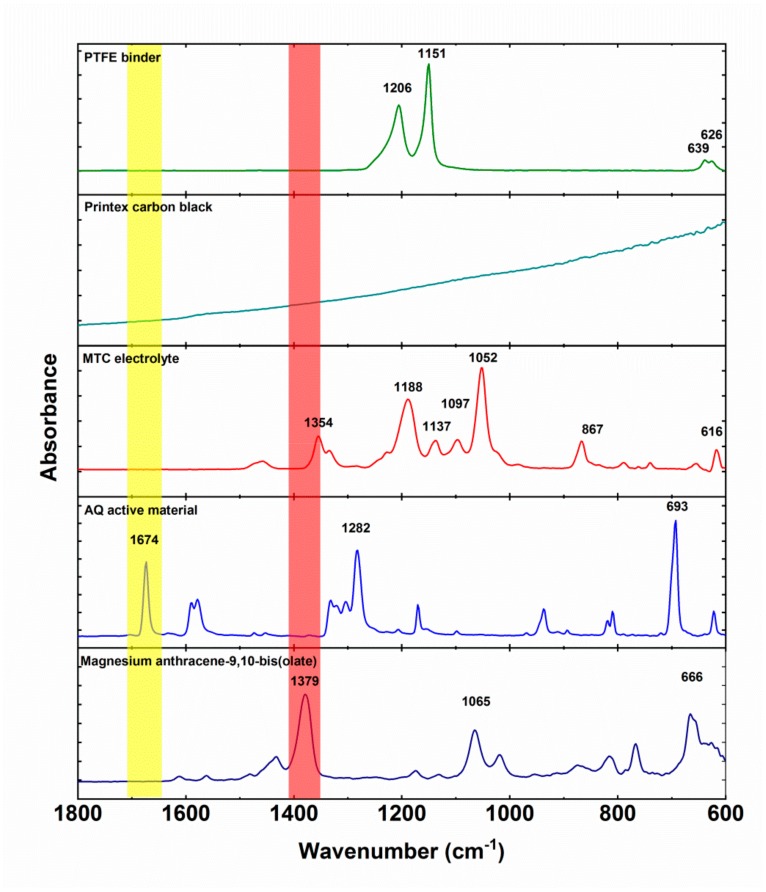
ATR-IR spectra of PTFE binder, Printex carbon black, AQ (charged active material), and magnesium anthracene-9,10-bis(olate) (discharged active material). All the measured compounds contribute to the spectra of ex situ electrodes.

In the last part of our work, we decided to investigate the electrochemical mechanism of our materials through ATR-IR. Although *operando* ATR-IR has shown to be extremely powerful, it is facing certain limitations when dealing with soluble species due to limited penetration depth and mixed response due to electrochemical activity and dissolution of active material [30]. Firstly, we measured the IR spectra of electrochemically inactive materials (Figure 5). Polytetrafluoroethylene binder displays two strong peaks at 1206 and 1151 and a smaller double peak at 639 and 626 cm^−1^. Printex carbon black does not exhibit any specific peaks, only strongly inclined background. MTC electrolyte has several strong peaks at 1354, 1188, 1137, 1097 1052, 867, and 616 cm^−1^. These peaks are a combination of all the components of the electrolyte (solvent, salts) and their interactions. Anthraquinone characteristic peaks are: Carbonyl peak at 1673 cm^−1^, ring vibration peak at 1282 cm^−1^, and out of plane vibraton at 693 cm^−1^. Magnesium anthracene-9,10-bis(olate) exhibits a ‒C‒O^−^ Mg^2+^ peak at 1379 cm^−1^, which is a slight upshift from ‒C‒O^−^ Li^+^ position observed previously [31] and consistent with the differences observed during *operando* ATR-IR measurements of anthraquinone polymers in Li and Mg batteries [30,31].

**Figure 6:**
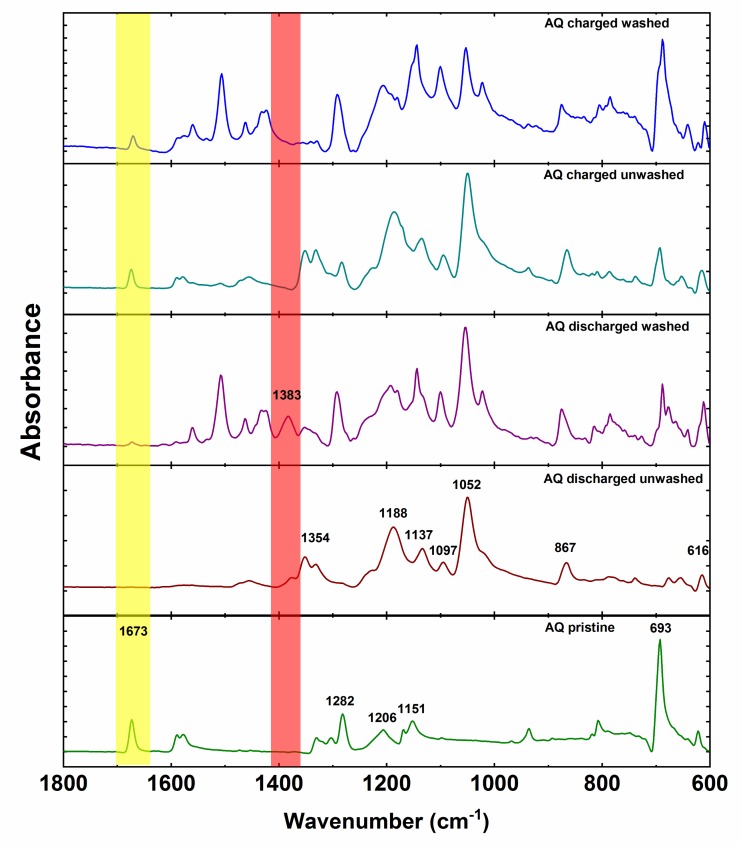
ATR-IR spectra of the AQ ex-situ electrodes.

Anthraquinone cathode exhibits all characteristic peaks of anthraquinone with the addition of PTFE peaks at 1206 and 1151 cm^−1^ (Figure 6). Spectra of ex situ unwashed electrodes are primarily dominated by the IR features of the electrolyte. However, unwashed discharged electrode displays an additional small side peak at 1383 cm^−1^ adjacent to the intense electrolyte peak at 1354 cm^−1^. After washing, the peak at 1383 cm^−1^ becomes more prominent while the intensity of the electrolyte peak decreases. This is in good agreement with the peak of pure magnesium anthracene-9,10-bis(olate). However, there is a difference of 4 cm^−1^ between the bands of pure synthesized compound and band of the electrochemically discharged AQ. This difference could be caused by tetrahydrofuran coordination of Mg in the case of synthesized magnesium anthracene-9,10-bis(olate). Interestingly, washing also reveals low-intensity AQ carbonyl peak, which confirms that indeed practical capacity utilization is not 100%. As in the case of the discharged electrode, also in the case of unwashed charged electrode, most of the peaks belong to the electrolyte. Nevertheless, carbonyl peak of AQ is relatively isolated and can be already easily seen in the unwashed electrode confirming reversible formation of carbonyl bond during charge. After washing, the peak at 693 cm^−1^ becomes the most intense in the spectrum and intensity of the electrolyte peaks lowers. Unfortunately, ex situ IR characterization of NQ and BQ did not give meaningful results due to the high solubility of NQ and BQ. Spectra of unwashed electrodes were dominated with electrolyte bands, and already brief washing of electrodes with DME solvent removed all the signals of the material, and only bands of the binder could be observed. Nevertheless, given the similar electrochemical behavior we can hypothesize that the electrochemical mechanism undergoes a reduction of the carbonyl bond during discharge and reversible formation of the carbonyl bond upon charge also in NQ and BQ. However, a more detailed investigation should be carried out using less soluble NQ and BQ derivatives.

## 4. Conclusions

Application of NQ and BQ as cathode-active materials has been somehow neglected in recent years due to the high solubility of these organic compounds in electrolytes and also limited oxidative stability of available Mg electrolytes. However, the energy density of Mg‒NQ and Mg‒BQ cells is 45% and 135% higher than the energy density of Mg‒AQ cell, respectively. This makes their application highly interesting for future rechargeable Mg metal–organic batteries. Their reversible redox potentials in Mg(TFSI)_2_-2MgCl_2_ electrolyte are in good agreement with predictions from the Li-organic battery analogy. The redox potential of all tested compounds in MgCl_2_-AlCl_3_ electrolyte displayed an upshift of electrochemical potential in the presence of AlCl_3_ species, which generalizes the effect previously observed on AQ based polymers to other quinone compounds. Ex situ ATR-IR characterization of cathodes was possible only for AQ due to the high solubility of NQ and BQ. In the discharge of AQ we were able to visualize the formation of ‒C‒O^‒^ Mg^2+^ band at 1383 cm^−1^. This peak disappeared during the charge and reversible formation of AQ carbonyl peak at 1673 cm^−1^ was observed, confirming the electrochemical mechanism of AQ in Mg batteries. Our work serves also as a call to polymer chemists to devise new polymerization procedures for the preparation of NQ and BQ based polymers if such high energy densities are to be realized in practical batteries. However, higher redox potentials of NQ and BQ will most likely require modified polymerization methods [23]. Continuous development of new organic polymers should be accompanied by the development of new Mg electrolytes with improved electrochemical properties (higher oxidative stability, non-corrosive…). Although for most of the current electrolytes’ oxidative stabilities well over 3.0 V are claimed, experimental tests often show much lower stabilities in practical battery cells. Thus, we propose additional tests with the cycling of carbon black in extended voltage windows that should enable a more realistic estimate of potential voltage windows. A combination of polymer cathodes based on NQ and BQ groups with next-generation Mg electrolytes should lead to practical high energy density Mg metal-organic batteries.

## Figures and Tables

**Table 1:** Theoretical cathode capacities, voltages, and energy densities for different metal‒organic batteries employing Li, Mg, and Al metal as anodes and anthraquinone, 1,4-naphthoquinone and 1,4-benzoquinone as cathodes. Energy values are calculated solely based on the mass of active materials. Voltages of Mg- and Al-organic cells are assessed from the voltage of Li cells and differences in the redox potentials of metals [15,21,22].

Cathode Material	Anthraquinone	1,4-Naphthoquinone	1,4-Benzoquinone
Cathode capacity	257 mAh/g	339 mAh/g	496 mAh/g
Voltage vs. Li	2.2 V	2.4 V	2.7 V
Cell energy density (Li)	530 Wh/kg	748 Wh/kg	1187 Wh/kg
Voltage vs. Mg	1.5 V	1.7 V	2.0 V
Cell energy density (Mg)	345 Wh/kg	500 Wh/kg	810 Wh/kg
Voltage vs. Al	0.8 V	1.0 V	1.3 V
Cell energy density (Al)	189 Wh/kg	304 Wh/kg	553 Wh/kg
